# Results from the Hawaii domestic violence fatality review, 2000-2009

**DOI:** 10.5249/jivr.v6i2.473

**Published:** 2014-07

**Authors:** Ann Pobutsky, Melissa Brown, Lisa Nakao, Florentina Reyes-Salvail

**Affiliations:** ^*a*^State Department of Health, Hawaii, US.; ^*b*^College of Public Health, University of Kentucky, US.; ^*c*^State Department of Human Services, Hawaii, US.

**Keywords:** Domestic violence, Fatalities, Hawaii

## Abstract

**Background:**

Patterns of domestic violence fatalities and agency responses in Hawaii have not been explicated.

**Methods:**

Retrospective reviews of events leading up to domestic violence related fatalities in Hawaii were assessed from 45 adjudicated cases that resulted in 62 fatalities for the ten year period from 2000-2009.

**Results:**

Almost one-half of the fatalities were homicide/suicide combinations. Females were disproportionately more likely to be fatal victims of domestic violence relative to their proportion in the population. Those aged 21-40 years and those over 80 years were more likely to be fatal victims of domestic violence, relative to their proportion in the population. Filipinas and ‘Other” ethnic groups are disproportionately more likely to be fatal victims of domestic violence while Native Hawaiians and Japanese are less likely to be fatal victims, relative to their proportions in the population. In more than two-thirds of the cases, the victim had made some attempt to leave the relationship prior to the fatality.

**Conclusions:**

In the majority of cases there was agency involvement in some form: either the victim alone or the perpetrator alone, or both. However, less than one-third (31.1%) of the cases over the past ten years had documentation of prior violence from medical reports, so this may be an area to further document and address domestic violence.

## Introduction

Women in the United States experience more intimate partner violence than do men. Estimates from the National Violence Against Women Survey show that 22.1 percent of surveyed women, compared with 7.4 percent of surveyed men, reported they were physically assaulted by a current or former spouse, cohabiting partner, boyfriend or girlfriend, or date in their lifetime. ^1^ In Hawaii it is estimated that about 132,000 adults (15%) experienced intimate partner violence in their lifetime, ^2^ however, only a few of those adults reported such experiences to authorities and even fewer resulted in fatalities. A substantial proportion of all homicides in the U.S. are committed by intimate partners of the victims, ^3,4^ with about 1 in 3 of female homicides committed by intimate/domestic partners. In the majority of cases of intimate partner/domestic violence fatalities (70-80%), no matter which partner was killed, the man physically abused the woman prior to the homicide.^4^ Nationally in 2008, Hawaii ranked #23 with a rate of 1.26 per 100,000 population of females murdered by males in single victim/single offender homicides, compared to 2.96 per 100,000 in Nevada (the highest). ^5^

In 1997, Hawaii Revised Statutes, HRS §321-471^6^ authorized the Department of Health (DOH) to conduct multidisciplinary and multiagency reviews of domestic violence fatalities to reduce the incidence of preventable fatalities. These retrospective reviews of events leading up to a domestic violence fatalities analyze (1) incident cases, their characteristics, risk factors and (2) the system responses to these cases by community agencies, institutions, and other organizations involved. The purpose of the Hawaii Domestic Violence Fatality Review (DVFR) is to conduct retrospective reviews of events leading up to a domestic violence fatality on a case by case basis. The review is nonjudgmental and is not to place blame on any of the agencies and entities involved in any of the cases prior to the fatality. The goal of reviewing domestic violence fatalities through fatality case reviews is similar to reviews conducted after airplane crashes: to help determine what went wrong and see what could have been done differently to prevent such fatalities. ^7^

The Hawaii Department of Health’s Domestic Violence Strategic Plan, 2007-2012 and the Hawaii Domestic Violence Action Center define domestic violence as a pattern of behavior which includes physical, sexual and/or emotional abuse between intimate partners (including verbal abuse and/or psychological tactics such as intimidation and/or degrading someone), including dating violence. Sometimes it involves other family members or friends. Domestic violence can include and can limit the ability of the victim to make personal choices, access family resources or assets and have self-determination.

**Domestic Violence Fatality Review Council and Team**

Selection and identification of cases for review is determined by the Domestic Violence Fatality Review (DVFR) Council.^8^ The DVFR Council is a multidisciplinary, multiagency group consisting of representatives from public and private agencies and organizations. Case sources can include newspaper articles, police reports, referrals from participating members, community agencies and institutions, the medical examiner’s office, and other sources. Other related fatalities secondary to the domestic violence fatality may also be reviewed on a case-by-case basis (e.g., neighbors, police officers, and other emergency workers). For example, sometimes a child or other family member becomes an additional victim in a domestic violence fatality. However, the separate Child Fatality Review (CDR) team reviews child fatalities resulting from domestic violence situations, although this does not preclude joint reviews with the Domestic Violence Fatality Review (DVFR) team. All cases are reviewed by a core DVFR team, whose members include representatives from the Medical Examiner’s Office, law enforcement, Department of Human Services, Emergency Medical Services, domestic violence advocates/coalition, the Prosecuting Attorney’s Office, the Department of Health and the Judiciary. The DVFR team travels throughout the state. At each fatality review, the team invites community/county representatives to participate.

**Case Ascertainment**

The DVFR team uses a broad definition of domestic violence fatality. Reviews may include family or household members as referenced in HRS §321-471. The reviews may include both homicides and suicides relating to domestic violence. Domestic violence fatalities include, but are not limited to: all homicides in which the victim was a current or former intimate partner of the perpetrator; homicides of people other than the intimate partner, which occur in the context of domestic violence or during an attempt to kill the intimate partner; homicides occurring as an extension of or in response to ongoing abuse between intimate partners, e.g., when an ex-spouse kills the children; and suicides that may be a result of domestic violence. 

The DVFR examines the facts and circumstances surrounding the fatality case under review. All information disclosed at the review is confidential. The formal review of a fatality is delayed until any criminal investigations or prosecutions connected with the fatality are completed. At the review, the facts and circumstances of the case are considered. This information is recorded on case review summary forms and includes: (1) background and basic demographics of the persons involved, (2) circumstances of the event, personal histories of the parties including any previous agency involvement: medical, mental health, financial, legal (civil and criminal complaints, e.g., arrests for assaults and/or abuse of family or household members, the existence of past or present protective orders), (3) services obtained by the victim, perpetrator and family prior to the fatal incident and services rendered after the fatality to family members and/or other affected persons and (4) outcomes. After the review, a determination is made by consensus as to whether or not the case is domestic violence related. The DVFR Team informs the DVFR Council on each review and the Council critically examines data and makes recommendations from each review to assure that system problems are addressed appropriately and timely, as well as follow up for improvement and change through training, policy development and/or legislative action. 

## Methods

Reviews of domestic violence fatalities occurring between the years 2000 and 2009 were conducted on all islands. There were 72 cases identified. Of these, 22 cases were not reviewed as these cases have not yet been adjudicated, which is a requirement of the DVFR. Of the remaining 50 cases, 45 were determined to be domestic violence related based on team determination after the review and 5 were found not to be domestic violence related.

Hard copies of all domestic violence related case fatality review summary forms (n=45 cases) were reviewed, variables created and data for each case were populated into a Statistical Package for the Social Sciences (SPSS) database in 2012. This database was specifically designed for this report and includes all variables on case review summary forms. The data were analyzed using SPSS and findings are summarized here. The data were analyzed first by total fatalities and then by cases, since almost one-half of the cases involved more than one fatality (homicide of the victim/suicide by perpetrator combinations as well as other fatalities). Data for all fatalities (n=62) were analyzed by characteristics of the victims and perpetrators (relationships), including sex, age ethnicity and county relative to the proportions in the population, and the circumstances of the fatalities (other additional fatalities and location of the fatalities). Data for the cases (n=45) were analyzed by characteristics, including demographics (sex, ethnicity, age at fatality, citizenship status and economic status), weapons used, attempts to leave by the fatality victim, agency involvement in the cases, fatality risk factors present, prior history of violence and the system responses to the cases. This information should inform and contribute to the analysis of system responses and recommend changes to prevent domestic violence fatalities. While this report represents 10 years of data, the number of fatalities is small. These small numbers limit statistical analysis and the ability to make definitive conclusions.

## Results

**Domestic Violence Related Fatalities (N=62)**

Table 1 provides a summary of the total domestic violence related fatalities reviewed from 2000-2009. The 45 domestic violence related reviewed cases resulted in 62 fatalities, of which 33 fatalities (53.2%) were female domestic violence victims, 31 of whom (50.0%) were killed by a current or former husband/boyfriend. There were 28 total fatalities (14 cases) which were combined homicide/suicides (45.2%). There were 7 fatalities (11.3%) among friends or family members of the domestic violence victim (one was a child of the victim). There were 5 male domestic violence victim fatalities (8.1%), 4 of whom (6.5%) were killed by their current or former partners (wife/girlfriend) and one suicide. Of the total fatalities (N=62), 37 or 60% involved only one death, whereas 25 or 40% involved two deaths. Overall, of the 45 domestic violence cases, 37 or 82% resulted in single fatalities (60% of all fatalities). However, of the multiple fatalities, a total of 25 deaths resulted from as few as 8 domestic violence cases. Four women were pregnant at the time of the fatality. One unborn child was delivered by cesarean section and survived, so there were an additional 3 fetal fatalities not included in total number of fatalities. 

**Table 1:** Total domestic violence related fatalities (N=62), Hawaii DVFR 2000-2009.

Fatalities	Killed by whom	#	%
Female DV victim	Current or former husband/boyfriend of DV victim	31	50.0%
Female DV victim	Female child abusing parent	1	1.6%
Female DV victim	Accident, but male DV perpetrator present	1	1.6%
Male perpetrator	Male child of female DV victim	1	1.6%
Male perpetrator	Female DV victim not in self defense	1	1.6%
Male perpetrator	Law enforcement	1	1.6%
Male perpetrator	Suicide	14	22.6%
Friend or family of female DV victim (includes 1 new male partner of DV victim) (5 female, 2 male)	Current or former husband/boyfriend of DV victim	7	11.3%
Male DV victim	Current or former wife/girlfriend of DV victim	4	6.5%
Male DV victim	Suicide	1	1.6%
**Total Fatalities**		62	100.0%
**Homicide/Suicide combination**		28	45.2%

Table 2 provides a summary of the total domestic violence fatalities by sex and age along with the average population in the State of Hawaii from 2000-2009, the population proportion, the number and proportion of fatalities. Females are disproportionately more likely to be fatal victims of domestic violence (61.3%) and men are less likely (38.7%), relative to their proportion in the population. In the analysis of all fatalities, we are including all male fatalities as domestic violence related, since 15 of the 25 male fatalities (60%) were suicides; there were no female suicides. By age groups, the proportions in the age groups, 21-30, 31-40 and over 80 years (8.1%) are disproportionately represented as victims of domestic violence, relative to their Hawaii state population age distribution. The differences from the state’s population proportion were statistically significant for both the 21-30 and the 31-40 age groups (p <.05 ) (see Table 2).

**Table 2:** Distribution of Hawaii population and domestic violence related fatalities (N=62) by sex and age, Hawaii DVFR 2000-2009.

	Average population size 2000-2009	% of population	# Fatalities	% of Fatalities
**Sex**				
Male	612,501	50.0%	24	38.7%
Female	620,481	50.0%	38	61.3%
Total	1,232,982	100.0%	62	100.0%
**Age/Years**				
0-21	353,320	28.7%	---	---
21-30	130,886	10.6%	13	21.0% *
31-40	166,364	13.5%	18	29.0% *
41-50	191,846	15.6%	9	14.5%
51-60	171,769	13.9%	10	16.1%
61-70	103,563	8.4%	6	9.7%
71-80	72,094	5.8%	0	0.0%
>80	43,140	3.5%	5	8.1%
Total	1,232,982	100.0%	62	100.0%

Source: Annual population estimates for 2000-2009 provided by the Office of Health Status Monitoring, Hawaii State Department of Health from the Hawaii Health Survey for the State of Hawaii and averaged. *Statistically significant at p<.05.

Table 3 provides a summary of total domestic violence fatalities by ethnicity and county, along with the average population from 2000-2009, the proportion of the population, the number and proportion of fatalities. Filipinos (24.2%) and other ethnic groups (19.4%) (Which includes Samoan, other Pacific Islander, Black, Mexican and 6 with multiple ethnicities) were more likely to be fatal victims of domestic violence. These ethnic groups were disproportionately represented relative to their proportions in the population (24.2% vs. 15% and 19.4% vs. 9.6% respectively) and the proportion differences were statistically significant (p<.05). Native Hawaiians (9.7%) and Japanese (14.5%) were disproportionately less likely to be fatal victims of domestic violence, relative to their proportion in the population, and this was statistically significant for Native Hawaiians (p<.05).

**Table 3:** Distribution of Hawaii population and domestic violence related fatalities (N=62) by ethnicity, county and crude fatality rates, Hawaii DVFR 2000-2009.

	Average population size 2000-2009	% of population	# Fatalities	% of Fatalities	Crude fatality rate per 100,000
**County**					
Honolulu	872,049	70.7%	40	64.5%	4.6
Hawaii	162,324	13.2%	10	16.1%	6.2
Kauai	61,176	4.9%	6	9.7%	9.8
Maui	137,421	11.1%	6	9.7%	4.4
Total	1,232,982	100.0%	62	100.0%	5.0
**Race/Ethnicity**					
Native Hawaiian/ part Native Hawaiian	290,346	23.5%	6	9.7%*	2.1
Japanese	246,253	20.0%	9	14.5%	3.7
Filipino	184,341	15.0%	15	24.2%*	8.1
Other Asian	99,995	8.1%	7	11.3%	7.0
White	293,463	23.8%	13	21.0%	4.4
Others **	118,584	9.6%	12	19.4%*	10.1
Total	1,232,982	100.0%	62	100.0%	5.0

Source: Population estimates for 2000-2009 provided by the Office of Health Status Monitoring, Hawaii State Department of Health. * Statistically significant at p<.05). ** Includes Samoan, other Pacific Islander, Puerto Rican, Black, Mexican and 6 with multiple ethnicities.

As seen in Table 3, neighbor island counties have higher proportions more likely to be victims of domestic violence relative to their numbers in the population (especially Kauai), while the city and county of Honolulu and Maui county have lower proportions (64.5%) compared to the total proportion in the population (70.7%). 

More than one in ten cases included innocent bystanders, not just a domestic violence pair (11.3% of total fatalities). Forty six of the 62 domestic violence fatalities (74.2%) occurred in a home. Twenty five occurred in the mutual home of the victim and perpetrator (40.3% of all fatalities), 14 in the victim’s home (22.6%), 6 in the DV perpetrator’s home (9.7%), and 1 in the home of a family or friend (1.6%). Three occurred at the domestic violence victim’s workplace (4.8%), while 13 occurred in a public area (21.0%) such as a street, park or public building. Among the fatalities occurring in a home, 20 were in the bedroom (43.5%), 8 in the kitchen (17.4%), and 7 in the living room (15.2%).

**Domestic Violence Related Fatality Cases (N=45)**

Table 4 examines the demographic characteristics of the domestic violence victims and perpetrators in the relationship that led to the fatality (N=45). The majority of the victims were female (86.7%) and the majority of the perpetrators were male (86.7%). Filipinas had the highest proportion (28.9%) being the victims of domestic violence relationships, followed by Europeans/Whites (20.0%) and those with multiple ethnicities (13.3%). Among perpetrators, the majority were Filipino (22.2%) followed by whites, other ethnic groups, multiple ethnicities (17.8% each) and Japanese (15.6%). In just over one-half of the cases (53.2%), the victim was between 21-40 years of age at the time of death. The majority of both victims and perpetrators were US citizens (71.1%), although there were some documented immigrant/refugee and some tourist cases. Most of the victims were employed (60%), as were the perpetrators (40%), yet many of the perpetrators were unemployed (17.8%). Educational status was not presented because more than half of all the cases had missing information for educational status. Chi-square analysis found that only sex had a significant association with domestic violence status (victim and perpetrator) (p<.05).

**Table 4:** Demographic characteristics of domestic violence fatality cases (N=45).

	DV Victim		DV Perpetrator	
	#	%	#	%
**Sex **				
Female	39	86.7%	6	13.3%
Male	6	13.3%	39	86.7%
Total	45	100.0%	45	100.0%
**Ethnicity**				
Filipino	13	28.9%	10	22.2%
White	9	20.0%	8	17.8%
Native Hawaiian/Part-Hawaiian	5	11.1%	2	4.4%
Japanese	4	8.9%	7	15.6%
Other Asian	5	11.1%	2	4.4%
Other (Includes Other Pacific Islanders, Puerto Rican, Black and Mexican).	3	6.7%	8	17.8%
Multiple ethnicities	6	13.3%	8	17.8%
Total	45	100.0%	45	100.0%
**Age at death**				
0-20	1	2.2%	0	0%
21-30	12	26.6%	3	6.7%
31-40	12	26.6%	4	8.8%
41-50	7	15.6%	4	8.8%
51-60	5	11.1%	3	6.7%
61-70	5	11.1%	1	2.2%
71-80	0	0%	0	0%
>80	3	6.7%	2	4.4%
Not applicable	0	0%	24	53.3%
Total	45	100.0%	45	100.0%
**Citizenship status**				
US Citizen	32	71.1	32	71.1%
Documented immigrant/refugee	6	13.3	8	17.8%
Tourist	1	2.2	1	2.2%
Unknown	6	13.3	4	8.9%
Total	45	1.0	45	100.0%
**Economic status**				
Employed	27	60.0	18	40.0%
Unemployed	3	6.7	8	17.8%
TANF/Food stamps/SSI/STD	6	13.3	6	13.3%
Other	5	11.1	4	8.9%
Unknown	4	8.9	9	17.8%
Total	45	100.0%	45	100.0%

In addition to the characteristics noted above, there were children in the family unit in 88.9% of the cases (n=40) and 2 women were pregnant at the time of the fatality. There were 31 cases (68.9%) where children were present at the location of the fatality event. Two children were involved as attempted homicides (one was a homicide as noted in Table 4). In 11 cases multiple weapons were utilized during the fatality event, so that 59 weapons were used in 45 cases. A knife was utilized 20 times (33.9%), a gun 14 times (23.7%). In addition, blunt objects (11.9%), body parts (11.9%), and motor vehicles (10.3%) were used as the weapon. Other weapons use included tools, flammable liquid/light and caregiver neglect.

In 20 cases (44.4%) the domestic violence victim had successfully left the relationship (both physically and emotionally). In 15 of the cases (33.3%) the victim had made some attempt to leave. [Of these 15 cases (data not shown), in 6 cases (13.3%) the victim had emotionally left (broken-up, stated intent to leave) but was still physically present. In 9 cases (20.0%) the domestic violence fatality victim had tried to leave previously but was physically and emotionally present when the fatality event occurred]. Therefore, in 35 cases (77.8%) the victim had made some attempt to leave.^*^ This aligns with intimate partner violence research that it is the most dangerous time for victims, when they leave or try to leave. 

In two thirds of the cases (73.3%), there was agency involvement with both the victim and perpetrator. However, in the vast majority of cases (95.6%) there was agency involvement in some form: either the victim alone, the perpetrator alone or with both. In almost one-half of the cases (44.4%) there were 5 or more agencies involved in the case, including law enforcement (29 cases), the prosecutors’ office (20 cases), family court/judge (22 cases), district court/judge (4 cases), circuit court/judge (10 cases), substance abuse treatment and assessment (13 cases), mental health (8 cases), health care providers (24 cases), local hospitals (9 cases), religious organizations/churches (4 cases), protection order advocacy program (4 cases), and DHS (6 cases). Other agencies involved (22 cases) include: community or court based legal advocacy organizations, culturally specific organization, homeless shelter, sexual assault program, EMS, other non-specified social service agency involvement, and other non-specified domestic violence victim support services. Health care system involvement (health care providers, mental health providers, local hospitals and EMS) was present in 41 of the 45 cases (91.1%).

Table 5 shows risk factors related to agency documents (reports from law enforcement, court, medical and counseling/advocacy) or verbal reports/accounts (perpetrator made threats to kill or harm victim and/or children and/or family members) by anyone (the victim or others) about prior violence or abuse event(s). Thirty one agency reports (68.9% of cases) and 34 verbal accounts by victims or others (75.6% of cases) provide evidence of prior physical abuse of the victim or others by the perpetrator. Thirty one victim or other verbal accounts (68.9% of cases) and 23 agency reports (51.1% of cases) also clearly document prior emotional/psychological abuse of the victim by the perpetrator. Sixteen agency reports (35.6% of cases) and 19 verbal accounts by victim/others (42.2%) also verify that the perpetrator made threats to kill the victim, children, family or friends. In 17 cases (37.8%) agency reports indicate the victim tried to leave within the past year, along with 28 cases (62.2%) based on verbal accounts by the victims/others. Agency reports show 14 cases had separation violence (31.1%), along with 28 cases based on verbal accounts of the victim/others (62.2%). The perpetrator used or abused drugs or alcohol in 24 agency reports (53.3%) and 25 verbal accounts by victims/others (55.6%). In the vast majority of cases there was a known history of domestic violence in any legal report/document for 36 of 45 cases (80.0%) and verbal accounts by anyone (victim or others) in 42 of 45 cases (93.3%). Therefore, in more than 95.6% of the cases (43 out of 45), there was a known history of domestic violence prior to the fatality.

**Table 5:** DV fatality risk factors related to agency documented and/or verbally reported previous violence and abuse (N=45), Hawaii DVFR 2000-2009.

	# Agency Documents	% Agency Documents	# Verbal Reports by Victim or Others	% Verbal Reports by Victim or Others
**Separation/physical access**				
DV Victim tried to leave within the last year	17	37.8%	28	62.2%
Separation violence	14	31.1%	25	55.6%
DV perpetrator had access to the victim	25	55.6%	39	86.7%
**Threats of violence**				
DV perpetrator made threats to kill victim	21	46.7%	26	57.8%
DV perpetrator made threats to kill children, family members or friends	16	35.6%	19	42.2%
DV perpetrator made suicide threats or attempts	6	13.3%	9	20.0%
DV perpetrator threatened victim/other with weapon (i.e. knife, gun, blunt weapon)	16	35.6%	22	48.9%
**Physical or other violence/abuse**				
DV perpetrator physically abused victim/others (i.e. shove, hit, slap, kick, etc.)	31	68.9%	34	75.6%
DV perpetrator abused victim emotional-ly/psychologically	23	51.1%	31	68.9%
DV perpetrator threatened or actually harmed pets or property	11	24.4%	14	31.1%
DV perpetrator sexually abused victim	4	8.9%	6	13.3%
DV perpetrator isolated victim (i.e. control of daily activities, holding passport, etc.)	8	17.8%	12	26.7%
**Behavioral and other problems**				
DV perpetrator was obsessively jealous	13	28.9%	18	40.0%
DV perpetrator used or abused drugs/alcohol	24	53.3%	25	55.6%
DV perpetrator diagnosed with psychological problems	11	24.4%	15	33.3%
DV perpetrator involved in physical or sexual abuse of children	9	20.0%	13	28.9%
DV perpetrator had previous charges of assault (non-specific type)	22	48.9%	21	46.7%
DV perpetrator had previous charges of abuse (non-specific type)	25	55.6%	26	57.8%

Table 6 shows the risk factors related to a prior history of violence based on agency documented and/or verbally reported prior violence. Less than one-third (31.1%) had documentation of prior violence from medical reports and less than one-fourth (22.2%) from counseling or advocacy reports. In more than one-half of the cases there was documented prior history of violence from law enforcement reports (71.1%), and/or court documents (62.2%), and in the majority of cases, verbal reports by the victim (88.9%) or others (93.3%). In the vast majority of cases (95.6%) or 43 of 45 cases, there was documentation and/or verbal reports of prior violence by the perpetrator.

**Table 6:** DV fatality risk factors related to disability, language and substance abuse (N=45), Hawaii DVFR 2000-2009.

Risk Factor	Victim #	Victim %	Perpetrator #	Perpetrator %
**Disability**				
Physical Disability	2	4.4%	4	8.9%
Cognitive Disability	2	4.4%	2	4.4%
Mental Disability	3	6.7%	7	15.6%
**Language **				
First language English	31	68.9%	28	62.2%
First language Filipino	4	8.9%	7	15.6%
First language other	5	11.1%	4	8.9%
First language not English	9	20.0%	11	24.4%
First language unknown	5	11.1%	6	13.3%
Could not speak/vocalize English without needing a translator	5	11.1%	8	17.8%
**Substance abuse and mental illness**				
Affected by drugs or alcohol at the time of the fatality	8	17.8%	22	48.9%
History of substance abuse (drugs and/or alcohol)	13	28.9%	28	62.2%
History of mental illness	5	11.1%	18	40.0%

Table 7 shows the risk factors related to disability, language and substance abuse for the 45 cases. In some cases there was evidence of physical, cognitive or mental disability, but no discernible pattern. However, English proficiency/language other than English was an issue. English was not the first language for 9 victims (20%) and 11 perpetrators (25%) and these included Filipino and other languages. A substantial number of victims (11.1%) and perpetrators (17.8%) could not speak or verbalize in English without a translator. More than one-half of the perpetrators (28, or 62.2%) had a history of substance abuse (drugs and/or alcohol) as did 13 of the victims (28.9%). Further, 22 of the perpetrators (48.9%) and 8 of the victims (17.8%) were affected by drugs or alcohol at the time of the fatality. Eighteen of the perpetrators (40.0%) and 11.1% of victims had a history of mental illness.

**Table 7:** DV fatality risk factors related to a known prior history of violence (N=45), Hawaii DVFR 2000-2009.

Prior History of DV*	# Cases	% Cases
Per law enforcement reports	32	71.1%
Per court documents	28	62.2%
Per medical reports	14	31.1%
Per counseling /advocacy reports	10	22.2%
Per any reports/documents	36	80.0%
Per victim (not documents/reports)	40	88.9%
Per others (not documents/reports)	42	93.3%
Per anyone (not reports/documents)	42	93.3%
In any form	43	95.6%

*More than one possible.

Table 8 addresses the relationship of known homicide cases from the Uniform Crime Reports (UCR) to the Domestic Violence Fatality Review (DVFR). The UCR reports 267 total homicides for the years 2000-2009. However, as stated earlier, cases cannot be reviewed by DVFR until they have completed adjudication. Earlier years have a higher completion rate of reviewed versus pending review cases for DVFR. For this reason the comparison was split into 5 year increments. Note that 31% of the cases in the DVFR for 2000-2009 are still pending review, so the analyses in Table 8 (and in the rest of this report), are limited to the 45 reviewed cases.

**Table 8:** Relationship of DV fatalities to known homicide cases reported by UCR (N=267) and Hawaii DVFR 2000-2009 (N=45).

2000-2004	UCR	DVFR		2005-2009	UCR	DVFR
Murder Total	146	28		Murder total	121	17
Total IPV #	30	27		Total IPV #	27	16
Total IPV %	20.5%	96.4%		Total IPV %	22.3%	94.1%
C/C Honolulu #	99	21		C/C Honolulu #	83	7
C/C Honolulu % of total homicides	67.8%	14.40%		C/C Honolulu % of total homicides	68.6%	5.8%
DVFR % of C/C Honolulu homicides		21.2%		DVFR % of C/C Honolulu homicides		8.4%
Hawaii #	26	4		Hawaii #	23	5
Hawaii % of total homicides	17.8%	2.7%		Hawaii % of total homicides	19.0%	4.1%
DVFR % of Hawaii homicides		15.4%		DVFR % of Hawaii homicides		21.7%
Maui #	10	1		Maui #	8	3
Maui % of total homicides	6.8%	0.7%		Maui % of total homicides	6.6%	2.5%
DVFR % of Maui homicides		10.0%		DVFR % of Maui homicides		37.5%
Kauai #	11	2		Kauai #	7	2
Kauai % of total homicides	7.5%	1.4%		Kauai % of total homicides	5.8%	1.7%
DVFR % of Kauai homicides		18.2%		DVFR % of Kauai homicides		28.6%

Source: Uniform Crime Reports http://bjs.ojp.usdoj.gov/ucrdata/Search/Crime/State/RunCrimeStatebyState.cfm http://hawaii.gov/ag/cpja/main/rs/Folder.2005-12-05.2910/

Uniform Crime Reports^9^ identifies the perpetrator of the homicide. This allows the calculation of the proportion of homicides that are specifically Intimate Partner Violence (IPV) utilizing statistics from the spouse and girlfriend/boyfriend perpetrator category. This allowed comparison with the DVFR for IPV cases. Non-IPV cases could not be compared. From 2000-2004 the UCR reports 30 IPV cases (20.5% of all homicides) and DVFR reports 27 cases of IPV (96.4% of all DV cases reviewed). Thus, for years 2000-2004 DVFR identified, reviewed, and confirmed 27 of 30 reported IPV homicide cases (90.0%). For years 2005-2009 UCR reports 27 IPV cases (22.3% of all homicides) while DVFR reports 16 cases of IPV (94.1% of all cases reviewed). Thus, for the years 2005-2009 DVFR has identified, reviewed and confirmed 16 of 27 reported homicide cases (59.3%). 

Having verified that the majority (90.0%) of reviewed cases from 2000-2004 were IPV allows for the estimation of IPV’s portion of total homicides for the state of Hawaii and by county. For the state overall, it can be estimated that 1 of 5 homicides are domestic violence related fatalities. The IPV percentage of all homicides for the city and county of Honolulu is 21.2% (1 in 5 homicides are due to IPV). For the Neighbor Islands these percentages are slightly lower at 15.4% for Hawaii County, 10.0% for Maui County, and 18.2% for Kauai County. Intimate partner violence does not capture the fatalities of family, friends, and new partners that are also homicide victims. Child fatalities are reviewed by a separate process and would also not be included in IPV counts. Therefore, it is possible that the percentage of domestic violence related homicides is slightly higher than 20-22%.

Table 9 lists identified barriers in the fatality cases reviewed. Multiple barriers were identified for each fatality case. Power and control, lethality or stalking issues were found to be present in 24 cases (53.3%). These are also known as “red flags” or warnings that domestic violence may be present. Though these early warning signs were identified they did not prevent the fatalities from occurring. The need for agency protocol improvement or change was identified in 19 cases (42.2%) as well as law or policy change in 16 cases (35.6%). Lack of involvement from significant others (family, friends, co-workers, etc.) was found in 18 cases (40.0%). Cultural, language or religious involvement and sensitivity regarding domestic violence identification and intervention was identified in 17 cases (37.8%). 

**Table 9:** Barriers* identified by the DVFR teams (N=45), Hawaii DVFR 2000-2009.

Barriers	Barriers #	Barriers %
Power and control, lethality or stalking issues present	24	53.3%
Agency protocol improvement or change needed	19	42.2%
Lack of involvement of significant others (family, friends, co-workers)	18	40.0%
Improve cultural, language and religious involvement and sensitivity	17	37.8%
Law or Policy change needed	16	35.6%
Improve and increase early intervention	14	31.1%
Temporary Restraining Order (TRO) specific change needed	12	26.7%
Lack of resource knowledge	11	24.4%
Screening improvement needed	10	22.2%
Increase referrals to existing organizations or agencies	10	22.2%
Systems change needed	9	20.0%
Lack of services or interventions available	8	17.8%
Drug or alcohol abuse present	8	17.8%
Financial difficulties present	7	15.6%
Multiple risk factors present	6	13.3%
Mental illness present	6	13.3%
Improve and increase training for professionals involved in DV	5	11.1%
Increase public education and awareness	5	11.1%
Safety planning needed	5	11.1%
Victim refused services	3	6.7%

*More than one could be selected.

Table 10 lists recommendations identified by the DVFR teams that could prevent or assist with domestic violence fatality prevention. Improving and increasing training for professionals in domestic violence was recommended in every case (100.0%). Agency protocol improvement or change was recommended in 43 cases (95.6%) and systems change in 19 cases 942.2%). Increasing public awareness regarding domestic violence prevention and assistance for those in need was recommended in 42 cases (93.3%). The need to increase referrals to existing organizations or agencies was recommended in 30 cases (66.7%). Increasing cultural, language and religious involvement and sensitivity was recommended in 20 cases (44.4%). In addition, there is also national recommended screening for intimate partner violence for girls beginning at age 14 (due to increases in teen dating violence) and at all visits of pregnant women to healthcare providers (due to escalation of intimate partner violence during pregnancy).

**Table 10:** Recommendations* identified by the DVFR teams (N=45), Hawaii DVFR 2000-2009.

Recommendations	#	%
Improve and increase training for professionals in domestic violence	45	100.0%
Agency protocol improvement or change	43	95.6%
Increase public education and awareness	42	93.3%
Increase referrals to existing organizations or agencies	30	66.7%
Increase cultural, language and religious involvement and sensitivity	20	44.4%
Systems change	19	42.2%
Temporary Restraining Order (TRO) specific change	15	33.3%
Screening improvement	14	31.1%
Improve and increase early intervention	14	31.1%
Increase safety planning	11	24.4%
Law or Policy change	10	22.2%
Increase services or interventions	7	15.6%

*More than one could be selected.

* (Physical leaving was defined as not living in the same residence as the perpetrator. Emotional leaving was defined as having ended the relationship (breaking-up, separation, divorce).)

## Discussion

This report highlighted the fact that domestic violence may lead up to premature deaths and can include not only one victim death per case but multiple victims. The overall number of domestic fatalities may be considered small at less than .01% of the total deaths during this period (62 period deaths due to domestic violence/89,393 total period deaths).^10^ Yet this number is greater than CDC’s standard of 50 for a minimum sample size. Regardless of the small numbers, premature deaths due to violence have a substantial burden and impact on Hawaii’s population. This report is a beginning to provide better insight on the characteristics, circumstances and background to such events and thus may be able to suggest ways to prevent or minimize the occurrence of fatalities attributable to domestic violence. This report provides useful data that could be compared with other states and the nation on domestic violence fatalities. On the other hand, given Hawaii’s multicultural environment the generalizability of the results may be limited. In Hawaii, over the past ten years, Filipinos and Other ethnic groups are disproportionately more likely to be fatal victims of domestic violence, relative to their proportion in the population. It is possible that tolerance for domestic violence is influenced by socio-cultural factors and length of immigration and acculturation. Steinberg,^11^ who spent more than 40 years studying the Philippines, suggested that: “Personalism, smooth interpersonal relationships, and hierarchical structures are safety lids for the Philippine society. Filipinos are both friendly and tolerant, but the society also tolerates moments of violence. To run amok is an understandable behavior if an individual has been wronged or provoked sufficiently. Crimes of passion abound and revenge is, in Philippine terms, often an acceptable explanation of criminal behavior. Temporary, explosive anger at a personal affront is a way Filipinos expresses existential rage. Political violence, especially just prior to elections, is widely accepted and usually unpunished”.

On the other hand, regardless of cultural issues the statistically significant differences found by age are also instructive, whereby younger people 21-30 years and 31-40 years are disproportionately more likely to be fatal victims of domestic violence suggests something about the nature of intimate partner relationships at this age (sexual reproductive age), along with sexual jealousies, possessiveness and power and control issues. A known history of prior violence is the key element in all these cases. 

The sociological and criminological literature provides a conceptual framework for explaining criminal or deviant behavior such as domestic violence. That is, deviant behavior is the result of the same social processes that result in conformity: these are learned behaviors.^12,13^ People learn the values, norms and beliefs of conformity, deviance or criminality through socialization and interaction with others. People learn that is acceptable or not acceptable to act violently toward family members, spouses, girlfriends or boyfriends through exposure to their own immediate families, and among other families in their communities as well as the tolerance for acts of violence in the community at large. Suggesting that violent behaviors learned could imply that such behaviors can be un-learned and changed as well.

There is a definite need for (1) increased awareness of the problem of domestic violence, (2) actions to prevent such fatalities from occurring and (3) the promotion of healthy non-violent relationships. Less than one-third (31.1%) of the Hawaii cases over the past ten years had documentation of prior violence from medical reports, so this may be an area to further document domestic violence. In Hawaii, there is information on domestic violence assistance available in Filipino languages (Ilocano and Tagalog) because of the disproportionate impact this has had on the Filipino community in Hawaii. This is due both to advocacy in various Filipino communities and public health efforts on different islands to educate the community. Information/translation in other non-English languages is also available by law upon request at all health facilities. In addition, more data on intimate partner/domestic violence injuries from emergency department and hospital discharges is being examined to better ascertain the extent of the problem of domestic violence in Hawaii.
